# A Bayesian Stackelberg Game Approach to Remote State Estimation Under SINR-Based DoS Attacks with Incomplete Information

**DOI:** 10.3390/s26041272

**Published:** 2026-02-15

**Authors:** Di Deng, Peng Yi, Mingze Qi

**Affiliations:** 1 Department of Control Science and Engineering, Tongji University, Shanghai 201804, China; dengdi@tongji.edu.cn; 2 Shanghai Research Institute for Intelligent Autonomous Systems, Shanghai 201210, China; 3 College of Science, National University of Defense Technology, Changsha 410073, China; qimingze17@nudt.edu.cn; 4 School of Physical Science and Engineering, Tongji University, Shanghai 200092, China

**Keywords:** cyber–physical systems, DoS attacks, incomplete information, Stackelberg game

## Abstract

With limited energy constraints, the issue of transmission and interference strategies have received considerable critical attention in cyber–physical security. In this paper, for remote state estimation under signal-to-interference-plus-noise ratio-based denial-of-service (DoS) attacks, the Stackelberg game between the sensor and the attacker is investigated. To balance estimation performance and energy consumption, the two players determine the transmission power and interference power sequentially under an incomplete information structure where the sensor does not know the fading channel gain of the attacker exactly. The schedule problem over the infinite-time horizon is first formulated as a Markov decision process with finite state and action spaces. Then, a Bayesian Stackelberg game (BSG) is constructed by incorporating the probability information of the channel interference gain. Based on the definition of best-response, the solution of the BSG is presented and the existence of the Stackelberg equilibrium is proven. Furthermore, a Stackelberg Q-learning algorithm is used to obtain the optimal strategies for the two players. Numerical results demonstrate the effectiveness of the proposed game method when the sensor is unable to access an attacker’s channel gain information.

## 1. Introduction

Cyber–physical systems (CPSs), which integrate computational capabilities with physical processes, have found extensive application across a range of industrial operations and critical infrastructure [1]. However, unreliable wireless communication networks render these systems highly susceptible to malicious cyber attacks, such as false-data injection (FDI), eavesdropping, and denial-of-service (DoS) attacks, which pose severe threats to their operational security and reliability [2].

In CPSs, security against malicious attacks, especially DoS attacks, has become a critical concern [3,4]. Game theory has emerged as a widespread and powerful analytical tool for modeling such attacker–defender interactions [5]. However, most existing game-theoretic studies are built upon the Nash equilibrium concept, which assumes simultaneous decision-making among players [6]. This assumption does not adequately capture the hierarchical structure often present in security scenarios, where a defender typically acts first and an attacker responds accordingly. To address this, Stackelberg games have been introduced, providing a more realistic framework for sequential decision-making in layered security strategies [7,8]. Furthermore, the majority of existing Stackelberg models rely on the assumption of complete information, where all players have perfect knowledge of each other’s parameters and payoffs. This is often impractical in real-world communication environments because some critical attributes such as channel conditions, interference gains, or attacker capabilities may not be fully accessible to the defender. The lack of such information significantly affects the strategic design and performance of defensive measures. Although some recent studies have begun to address information asymmetry, their modeling of uncertainty remains limited. For instance, most of these works focus on single-type uncertainty or assume that the attacker’s channel gain follows a simple bivariate distribution [9]. Such simplifications fail to capture the practical situation where the attacker’s fading channel gain may follow a multi-type probability distribution.

Motivated by these gaps, this paper investigates remote state estimation under SINR-based DoS attacks within a Bayesian Stackelberg game framework. It is worth noting that our work focuses on the strategic response and resource allocation following the detection or presumption of an attack, rather than on the attack detection mechanisms themselves [10]. While Bayesian Stackelberg models have been studied for general network security, this work distinctively integrates this framework into the SINR-based remote state estimation to handle multi-type channel gain uncertainty and further develops a model-free Stackelberg Q-learning algorithm to derive optimal strategies under this specific information structure. In the incomplete information setting, probabilistic distribution information is incorporated to model the uncertainty of the attacker’s fading channel gain, and a sequential power allocation strategy is developed to balance estimation accuracy and energy efficiency. The proposed approach not only extends current Stackelberg-based security analysis to more realistic communication scenarios but also offers implementable learning-based strategies for deriving equilibrium policies under information asymmetry. The main contributions of this paper are summarized as follows:

1. We study the strategic interaction between a sensor and a DoS attacker under energy constraints in an SINR-based remote state estimation system with unknown channel gain information. The sequential decision-making process is formulated as a finite-state and finite-action MDP, capturing the dynamic and uncertain nature of the environment.
2. To address the incomplete information regarding the attacker’s channel gain, we model the interaction as a BSG, where the attacker is treated as having multiple possible types. Within this framework, we define the best-response strategies, prove the existence of an SE, and analytically derive the SE strategies for both players.
3. A Stackelberg Q-learning algorithm is used to enable both players to learn their optimal strategies without prior knowledge of the opponent’s payoff structure. This model-free approach ensures adaptability and practicality in real-world settings. Finally, numerical simulations validate the effectiveness and robustness of the proposed BSG-based method, demonstrating its clear advantage over conventional Nash-based approaches under information asymmetry.

The remainder of this article is organized as follows. Section 2 reviews related work on secure state estimation under cyber-attacks. Section 3 establishes the system model for remote state estimation under SINR-based DoS attacks with incomplete channel gain information. Section 4 formulates the sequential decision-making process as a two-player Markov Decision Process (MDP). Building upon the MDP, Section 5 constructs the Bayesian Stackelberg Game (BSG) framework and analyzes the Stackelberg equilibrium. Section 6 designs a Stackelberg Q-learning algorithm to compute the optimal strategies for both players. Section 7 provides simulation results to verify the effectiveness of the proposed approach. Finally, conclusions are drawn in Section 8.

## 2. Related Work

Accurate and secure state estimation serves as the cornerstone of reliable decision-making in CPSs. However, the widespread dependence on wireless networks for data transmission exposes the estimation process to various cyber attacks, including FDI, eavesdropping, and DoS attacks. These attacks compromise data integrity, availability, and confidentiality [11]. These attacks can deliberately degrade estimation accuracy by corrupting, intercepting, or blocking data transmissions, which, in turn, destabilize system operation and threaten physical security [12,13].

DoS attacks, especially those targeting the signal-to-interference-and-noise ratio (SINR) of wireless channels, are the most disruptive to remote state estimation [4,14,15]. The sensor, as the defender, aims to enhance the system performance with less transmission power. On the contrary, the goal of the DoS attacker is to reduce estimation accuracy while consuming more communication resources. To describe such adversarial dynamics between attackers and defenders, game theory has emerged as a powerful analytical tool for the interactive decision-making process [16,17,18,19]. With energy constraints for both the sensor and the attacker, a Nash equilibrium (NE) of a zero-sum game was proven in [20] to be the optimal strategies for both sides. Then, the authors in [21] proposed a a Markov game framework under the SINR-based DoS attacks and applied a modified Nash Q-learning algorithm to obtain the optimal solutions. For multi-channel networks, a two-player zero-sum stochastic game was formulated to design the mixed strategies for the channel selection of the sensor and DoS attacker [22].

It is worth pointing out that most works on attack–defense games adopt NE as the game solution based on the assumption that the defender and the attacker choose their actions simultaneously, which is not applicable for situations where the players deploy their strategies sequentially. Consequently, the Stackelberg game, with its explicit “leader–follower” structure, has been a more appropriate and realistic framework for capturing the hierarchical interactions [23]. For the SINR-based fading wireless network, a Stackelberg equilibrium (SE) within a two-player nonzero-sum Markov game framework was constructed in [24] to obtain the transmission and interference strategies for the sensor and attacker, respectively. By taking the Stackelberg game method, the linear quadratic Gaussian (LQG) control strategy was analyzed in [25,26] for FDI attacks and DoS attacks, respectively. For distributed Kalman filtering, a Stackelberg game-based distributed reinforcement learning algorithm was developed to produce joint optimal strategies based on local observation information [27]. Within the Stackelberg game framework, an optimal stealthy robust attack method was designed based on Wasserstein ambiguity sets [28].

The aforementioned Stackelberg-based studies rely on the assumption of complete information, which rarely holds in practical wireless communication scenarios. For players in games, some crucial environmental information and other’s key attributes are often difficult to obtain, such as energy budgets, acknowledgment (ACK) information, and channel gains of the wireless network. An anti-jamming Bayesian Stackelberg game was proposed in [29], where the uncertainties of the channel state information and transmission cost information were incorporated. For the problem of multi-channel power schedule SINR-based DoS attacks, the SE was studied under two types of incomplete information, where the existence of the attacker and the total power of the attacker were, respectively, unknown to the defender [9]. For a multi-hop network under DoS attacks, the defender had no access to the available energy of the attacker; then, a Bayesian Stackelberg game was implemented and a Stackelberg Q-learning algorithm was presented to obtain the SE [30]. Furthermore, a stochastic Bayesian game was formulated in [31], where the ACK information from the remote estimator to the sensor was hidden from the attacker. While game theory provides a robust framework for strategic analysis, it is also worth noting that formal methods have been extensively explored for the verification and analysis of CPSs under attacks [32]. In contrast to these verification-based approaches, this paper emphasizes the adaptive decision-making process in scenarios where the sensor faces incomplete information about an attacker’s characteristics.

## 3. Problem Formulation

*Notations:* $N_{0}$ denotes the set of nonnegative integers. $R^{n}$ and $R^{m\times n}$ are the *n*-dimensional Euclidean space and set of all m×n matrices, respectively. $S_{⪰0}^{n}$ and $S_{≻0}^{n}$ represent the sets of n×n real symmetric positive semi-definite and positive definite matrices, respectively. If $X\in S_{⪰0}^{n}$, we simply write X≥0 (X>0 if $X\in S_{≻0}^{n}$). Notation X<Y means that the matrix X−Y is negative definite. Pr(·) is the probability of a random event. E[·] and Cov[·] stand for the expectation and covariance of a random vector, respectively. For functions *g*, *h* with appropriate domains, g∘h(·) represents the function composition g(h(·)), with $h^{n}\left(\cdot \right)≜h\left(h^{n-1}\left(\cdot \right)\right)$.

**Figure 1:**
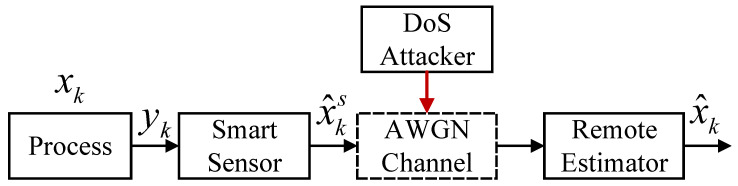
Remote state estimation over AWGN channel under DoS attacks.

In this section, we introduce the remote state estimation system under DoS attack depicted in Figure 1. The local sensor estimates the system states by the Kalman filter and then transmits the estimates to the remote estimator through the signal-to-noise ratio (SNR)-based network. However, the network is unreliable and may be attacked by a DoS attacker.

### 3.1. System and Sensor Model

Consider the discrete-time linear time invariant system:

(1) $$\begin{array}{cc} x_{k+1} & =Ax_{k}+w_{k}, \end{array}$$

(2) $$\begin{array}{cc} y_{k} & =Cx_{k}+v_{k}, \end{array}$$

where $x_{k}\in R^{n}$ is the system state and $y_{k}\in R^{m}$ is the measurement output. The process noise $w_{k}\in R^{n}$ and the measurement noise $v_{k}\in R^{m}$ are assumed to be independent white Gaussian noises with covariance matrices $Q\in S_{≻0}^{n}$ and $R\in S_{≻0}^{m}$, respectively. The initial state $x_{0}$ is zero-mean Gaussian with covariance $P_{0}^{-}\in S_{≻0}^{n}$, and independent of $w_{k}$ and $v_{k}$ for all $k\in N_{0}$. The pair (A,C) is detectable.

The smart sensor runs a local Kalman filter to estimate the system states based on the the measurement set up to the current time *k*, that is, $Y_{k}=\left\{y_{1},y_{2},\ldots ,y_{k}\right\}$. The local minimum mean-squared error (MMSE) estimate of the system state $x_{k}$ and the corresponding estimation error are, respectively, defined as

$$\begin{array}{cc} {\hat{x}}_{k}^{s} & =E[x_{k}|Y_{k}],\\ P_{k}^{s} & =E[\left(x_{k}-{\hat{x}}_{k}^{s}\right){\left(x_{k}-{\hat{x}}_{k}^{s}\right)}^{⊤}|Y_{k}]. \end{array}$$

We define the the Lyapunov and Riccati operators *h* and *g*: $S_{⪰0}^{n}\to S_{⪰0}^{n}$ as follows:

$$\begin{array}{cc} h(X) & ≜AXA^{⊤}+Q,\\ g(X) & ≜X-XC^{⊤}{\left[CXC^{⊤}+R\right]}^{-1}CX. \end{array}$$

The estimation error covariance of the Kalman filter converges to a unique value irrespective of the initial condition. For simplicity, it is assumed that the local Kalman filter has reached the steady state, and we let

(3) $$P_{0}^{s}=\bar{P},$$

where $\bar{P}$ is the steady-state error covariance given by the unique positive semidefinite solution of g∘h(X)=X.

### 3.2. Communication Channel and Attack Model with Incomplete Information

After obtaining the state estimate $x_{k}^{s}$, the sensor transmits the estimate to the remote estimator in the form of a data packet. However, random data packet drops may occur owing to channel fading and interference. Here, we assume that the sensor communicates with the remote estimator over an additive white Gaussian noise (AWGN) network to model this scenario. The packet dropout probability at time *k* is described by $SNR_{k}=\frac{\alpha \lambda_{k}}{\sigma_{k}}$, where α>0 is the fading channel gain of the sensor, $\lambda_{k}$ is the transmission power of the sensor at time *k*, and $\sigma_{k}$ is the additive white Gaussian noise power.

Considering a DoS attacker against the channel, the SNR is revised as the following SINR:

(4) $$SINR_{k}=\frac{\alpha \lambda_{k}}{\sigma_{k}+\beta \delta_{k}},$$

where β>0 is the fading channel gain of the attacker; $\delta_{k}$ is the transmission power of the attacker at time *k*.

The transmission of $x_{k}^{s}$ between the sensor and the remote estimator can be characterized by a binary random process $\left\{\gamma_{k}\right\}$:

$$\gamma_{k}=\left\{\begin{array}{cc} 1, & \mathrm{if}\;x_{k}^{s}\mathrm{is}\;\mathrm{received}\;\mathrm{successfully},\\ 0, & \mathrm{otherwise}. \end{array}\right.$$

Then, the packet arrival rate $\mu_{k}$ is given as

(5) $$\mu_{k}≜\mathrm{Pr}\left(\gamma_{k}=1\right)=1-2Q\left(\sqrt{\kappa SINR_{k}}\right),$$

where κ>0 is a parameter; $Q\left(x\right)=\frac{1}{\sqrt{2\pi }}\int_{x}^{\infty }\exp\left(-\frac{u^{2}}{2}\right)du$ represents the standard *Q*-function. It should be noted that the scalar function Q(·) here is distinct from the system noise covariance matrix *Q* defined in the system models (1) and (2). It is seen that the SINR is not only dependent on the sensor’s transmission power but is also influenced by the interference power generated by the DoS attacker. Obviously, the larger SINR leads to the lower packet loss rate and better estimation performance.

In practice, the channel state information is not perfect. The uncertainties of the channel gain information need to be considered. Suppose that the attacker interferes with the transmission under channel gain $\beta_{j}$ with probability $\eta (\beta_{j})$, where j∈N={1,2,…,H} and $\sum_{j=1}^{H}\eta \left(\beta_{j}\right)=1,H\geq 2$. Then, the SINR with the channel interference gain $\beta_{j}$ is defined as

(6) $$SINR_{k}^{j}=\frac{\alpha \lambda_{k}}{\sigma_{k}+\beta_{j}\delta_{k}^{j}}.$$

Correspondingly, the packet arrival rate under ${\mathrm{SIN}R}_{k}^{j}$ is given as

(7) $$\mu_{k}^{j}≜\mathrm{Pr}\left(\gamma_{k}=1\right)=1-2Q\left(\sqrt{\kappa SINR_{k}^{j}}\right).$$

This multi-type probabilistic model captures a realistic scenario where the sensor, as a defender, cannot precisely identify the attacker’s equipment or instantaneous channel condition but can estimate a probability distribution over a few distinct levels of threat severity. In this case, the sensor does not know the channel interference gain of the attacker but possesses the probability distribution of β. Therefore, with different levels of channel interference gain, we can think that there exists *H* type attackers in the environment.

**Remark** **1.** *In practice, the acquisition of channel information often has a non-symmetric characteristic. An attacker can frequently intercept the sensor’s open pilot signals or reference transmissions. Then, the attacker can accurately estimate the sensor’s transmission channel gain by processing these known sequences [33,34]. When launching an active attack, the attacker employs non-cooperative, noise-like, or protocol-aware jamming signals, which are deliberately designed to be unpredictable [35]. Thus, it is difficult for the sensor to accurately estimate the interference channel gain from the attacker.*

### 3.3. Remote State Estimation

The MMSE estimate of $x_{k}$ and the estimation error covariance at the remote estimator are denoted as ${\hat{x}}_{k}$ and $P_{k}$, respectively. According to whether the date is received successfully, the state estimation is given by

(8) $${\hat{x}}_{k}=\left\{\begin{array}{cc} {\hat{x}}_{k}^{s}, & \gamma_{k}=1,\\ Ax_{k-1}, & \gamma_{k}=0. \end{array}\right.$$

As a result, the error covariance $P_{k}$ is computed as

(9) $$P_{k}=\left\{\begin{array}{cc} \bar{P}, & \gamma_{k}=1,\\ h(P_{k-1}), & \gamma_{k}=0. \end{array}\right.$$

Assume that the initial value of the error covariance at the remote estimator also starts from $\bar{P}$, i.e., $P_{0}=\bar{P}$. The remote estimator will send ACKs to the sensor to indicate whether it has received the estimate at each time. Hence, the sensor can also calculate $P_{k}$ by (9). Note that $P_{k}$ takes values from the infinitely set $\left\{\bar{P},h\left(\bar{P}\right),h^{2}\left(\bar{P}\right),\ldots \right\}$.

### 3.4. Strategy and Objective Function

Considering that both the sensor and the attacker have power constraints, we assume that the transmission power and attack power take values from the finite sets. Specifically, the transmission power $\lambda_{k}$ belongs to a finite set with level $l_{d}$, which is denoted as $\Lambda =\{\lambda_{1},\ldots ,\lambda_{l_{d}}\}$. The interference power $\delta_{k}^{j}$ belongs to a finite set with level $l_{a}^{j}$, which is denoted as $\Delta^{j}=\left\{\delta_{1}^{j},\ldots ,\delta_{l_{a}^{j}}^{j}\right\}$. This discretization models the practical constraints of digital power amplifiers and energy-limited devices and is a common simplification in power control problems.

The strategies of the sensor and the *j*-th type attacker at time *k* are $\lambda_{k}\in \Lambda$ and $\delta_{k}^{j}\in \Delta^{j}$ for all $k\in N_{0}$, respectively. Then, the strategy of the attacker at time *k* is denoted as

$$\delta_{k}=\left\{\delta_{k}^{1},\delta_{k}^{2},\ldots ,\delta_{k}^{H}\right\}.$$

From (6) and (7), different types of attacker lead to different packet arrival rates and further affect the expected estimation error. The trace of the expected estimation error covariance under the attacker’s channel gain $\beta_{j}$ is given as

(10) $$f_{k}^{j}≜E\left[\mathrm{tr}\left(P_{k}^{j}\right)\right]=\mathrm{tr}\left(\mu_{k}^{j}\bar{P}+\left(1-\mu_{k}^{j}\right)h\left(P_{k-1}\right)\right).$$

The sensor aims to optimize the expected estimation error while reducing the total transmission cost. In contrast, the goal of the attacker is to deteriorate the estimation performance of the remote estimator and consume as much transmission energy of the sensor as possible while minimizing the total attack cost. Therefore, the one-step rewards of the sensor and the attacker with channel gain $\beta_{j}$ are, respectively, given as

(11) $$\begin{array}{cc} r_{k,d}\left(\lambda_{k},\delta_{k}\right) & =-\sum_{j=1}^{H}\eta \left(\beta_{j}\right)f_{k}^{j}-C_{d}\lambda_{k}, \end{array}$$

(12) $$\begin{array}{cc} r_{k,a}^{j}\left(\lambda_{k},\delta_{k}^{j}\right) & =f_{k}^{j}+C_{d}\lambda_{k}-C_{a}\delta_{k}^{j}, \end{array}$$

where $C_{d}$ and $C_{a}$ are the costs per unit power for the sensor and DoS attacker, respectively. Then, the corresponding payoff functions over the infinite time horizon for the sensor and the *j*-th type attacker are, respectively, given as follows:

(13) $$\begin{array}{cc} J_{d}\left(\lambda_{k},\delta_{k}\right) & =\sum_{k=1}^{\infty }\rho_{k-1}r_{k,d}\left(\lambda_{k},\delta_{k}\right), \end{array}$$

(14) $$\begin{array}{cc} J_{a}^{j}\left(\lambda_{k},\delta_{k}^{j}\right) & =\sum_{k=1}^{\infty }\rho_{k-1}r_{k,a}^{j}\left(\lambda_{k},\delta_{k}^{j}\right), \end{array}$$

where ρ∈(0,1) is the discount factor.

For the considered system under attack, the sensor as the defender first decides its transmission power level and then the DoS attacker chooses the interference power level based on the current situation. For such sequentially interactive decision-making processes with incomplete information, we use the Bayesian Stackelberg game framework to analyze the optimal strategies for the sensor and attacker.

**Remark** **2.** *Existing studies on SINR-based attacks against state estimation predominantly design transmission and interference strategies under the assumption of complete channel information [20,21,22,26]. This idealization ignores the reality that channel knowledge is incomplete and asymmetric in actual wireless networks, leading to multi-modal players and their decision-making spaces. Therefore, investigating the resulting hierarchical game is both highly meaningful and challenging.*

## 4. MDP Formulation

In this section, a Markov decision process is first formulated to describe the dynamics of the state estimation at the remote estimator. Obviously, the scenario involves two players: the sensor and DoS attacker. The sensor does not know the attacker’s channel gain but has knowledge of the probability distribution information. At each time, the sensor and attacker take action based on the current process state and the information that they have previously collected. Then, they respectively receive the rewards, and the process moves to the next state according to the transition probability.

Taking into account the decision-making interaction between the sensor and the attacker, the state estimation process at the remote estimator can be formulated as a two-player MDP model, which consists of the following five essential elements:

(1) *Desion epoch:* Let *T* denote the infinite discrete set of decision epochs, i.e., T={1,2,…}.

(2) *State:* According to (9), the estimation error covariance can be alternatively written as $P_{k}=h^{\tau_{k}}\left(\bar{P}\right)$, where $\tau_{k}=\left\{1,2,\ldots \right\}$ is the holding time at time *k*, namely, the time interval between the current time *k* and the latest moment of the receiving packet. Then, the set of the possible estimation error covariance $P_{k}$ can be represented as $Z_{k}=\left\{\bar{P},h\left(\bar{P}\right),\ldots ,h^{k}\left(\bar{P}\right)\right\}$. Therefore, the state at time *k* is defined as the holding time of the time k−1, i.e., $s_{k}=\tau_{k-1}$. Intuitively, this state $s_{k}$ represents the time interval elapsed since the last successful packet reception. According to the update Formula (9), it directly determines the current estimation error covariance at the remote estimator. Due to the low probability of a large holding time, the final state *K* is used to represent all states with $\tau_{i}\geq K$. Therefore, the state space is S={0,1,…,K}.

(3) *Action:* At each time, based on the state $s_{k}$, the sensor and the *j*-th type attacker choose the actions $\lambda_{k}$ and $\delta_{k}^{j}$ from the action spaces $A_{d}=\Lambda$ and $A_{a}^{j}=\Delta^{j}$, respectively.

(4) *Transmission probability:* Given the action $\lambda_{k}$ of the sensor and the action $\delta_{k}^{j}$ of the *j*-th type attacker, the probability that the state transmits from $s_{k}$ to $s_{k+1}$ is given as μ.

(15) $$\mathrm{Pr}\left(s_{k+1}|s_{k},\lambda_{k},\delta_{k}^{j}\right)=\left\{\begin{array}{cc} \mu_{k}^{j}, & s_{k+1}=0,\\ 1-\mu_{k}^{j}, & s_{k+1}=s_{k}+1,\\ 0, & \mathrm{otherwise}. \end{array}\right.$$

(5) *Reward:* The one-stage reward functions for the sensor and the *j*-th type attacker are, respectively, given as

(16) $$\begin{array}{cc} & r_{k,d}\left(s_{k},\lambda_{k},\delta_{k}\right)\\ = & -\sum_{j=1}^{H}\eta \left(\beta_{j}\right)\mathrm{tr}\left[\mu_{k}^{j}\bar{P}+\left(1-\mu_{k}^{j}\right)h^{s_{k}+1}\left(\bar{P}\right)\right]-C_{d}\lambda_{k}, \end{array}$$

and

(17) $$\begin{array}{cc} & r_{k,a}^{j}\left(s_{k},\lambda_{k},\delta_{k}^{j}\right)\\ = & \mathrm{tr}\left[\mu_{k}^{j}\bar{P}+\left(1-\mu_{k}^{j}\right)h^{s_{k}+1}\left(\bar{P}\right)\right]+C_{d}\lambda_{k}-C_{a}\delta_{k}^{j}. \end{array}$$

Note that as the one-stage reward functions are time-invariant and stationary and can also be denoted as $r_{d}\left(s_{k},\lambda_{k},\delta_{k}\right)$ and $r_{a}^{j}\left(s_{k},\lambda_{k},\delta_{k}^{j}\right)$.

The sensor’s strategy $\pi_{d}\left(s\right)=\lambda_{k}\left(s\right)$ means that the sensor takes action $\lambda_{k}$ under state $s_{k}$. Similarly, the strategy of the *j*-th type attacker under state *s* is denoted as $\pi_{a}^{j}\left(s\right)=\delta_{k}^{j}\left(s\right)$. Then, the strategies of the sensor and the *j*-th type attacker are, respectively, written as

$$\begin{array}{cc} \pi_{d} & =\left\{\pi_{d}\left(s\right)|\forall s\in S\right\}\in \Pi_{d},\\ \pi_{a}^{j} & =\left\{\pi_{a}^{j}\left(s\right)|\forall s\in S\right\}\in \Pi_{a}^{j}, \end{array}$$

where $\Pi_{d}$ and $\Pi_{d}^{i}$ are the set of all stationary and deterministic policies for the sensor and *j*-th type attacker, respectively. Furthermore, the strategy of the attacker is denoted as

$$\pi_{a}=\left[\pi_{a}^{1},\pi_{a}^{2},\ldots ,\pi_{d}^{H}\right]\in \Pi_{a}$$

where $\Pi_{a}$ is the set of all stationary and deterministic policies for the attacker. Then, the payoff functions of the sensor and *j*-th type attacker are, respectively, given as

(18) $$\begin{array}{cc} J_{d}\left(s,\pi_{d},\pi_{a}\right) & =\sum_{k=1}^{\infty }\rho^{k-1}r_{d}\left(s_{k},\lambda_{k},\delta_{k}\right), \end{array}$$

(19) $$\begin{array}{cc} J_{a}^{j}\left(s,\pi_{d},\pi_{a}^{j}\right) & =\sum_{k=1}^{\infty }\rho^{k-1}r_{a}^{j}\left(s_{k},\lambda_{k},\delta_{k}^{j}\right). \end{array}$$

The goal of both the sensor and the attacker is to seek the optimal strategy to maximize the payoff function.

## 5. Bayesian Stackelberg Game

Based on the above MDP framework, a BSG with two players was investigated for designing the optimal strategies for both the sensor and the attacker. In this hierarchical game, the sensor, as the leader, first makes its action according to the state and declares its strategy. It is noted that the sensor knows the reaction from the attacker in a Stackelberg game. Then, the attacker, as the follower, takes action based on the acquired channel gain and the transmission power of the sensor. The payoff functions of the sensor and *j*-th type attacker are given as (18) and (19), respectively. The SE of the BSG is analyzed and the corresponding optimal strategies for both players are provided. The best response for each side is first defined as follows.

**Definition** **1.** *The best response is that a player takes an action that optimizes its own payoff while taking into account other players’ actions. Specifically, the best responses for the sensor and j-th type attacker are given as*

(20) $$\begin{array}{cc} \phi_{d}^{*}\left(\pi_{a}\right) & =\arg\max_{\pi_{d}\in \Pi_{d}}J_{d}\left(s,\pi_{d},\pi_{a}\right), \end{array}$$

(21) $$\begin{array}{cc} \phi_{a}^{j,*}\left(\pi_{d}\right) & =\arg\max_{\pi_{a}^{j}\in \Pi_{a}^{j}}J_{a}^{j}\left(s,\pi_{d},\pi_{a}^{j}\right),\;j\in N. \end{array}$$

*Then, the best responses for the attacker are denoted as*

(22) $$\phi_{a}^{*}\left(\pi_{d}\right)=\left[\phi_{a}^{1,*}\left(\pi_{d}\right),\phi_{a}^{2,*}\left(\pi_{d}\right),\ldots ,\phi_{a}^{H,*}\left(\pi_{d}\right)\right].$$

The best-response set of the *j*-th type attacker to the sensor’s strategy $\pi_{d}$ is denoted as $R_{a}^{j}\left(\pi_{d}\right)$ and $R_{a}\left(\pi_{d}\right)=\left[R_{a}^{1}\left(\pi_{d}\right),\ldots ,R_{a}^{H}\left(\pi_{d}\right)\right]$ is the best-response set of the attacker to strategy $\pi_{d}$.

**Lemma** **1.** *For the proposed BSG with two players, the Stackelberg equilibrium $\pi_{d}^{SE}$ of the sensor satisfies*

(23) $$J_{d}\left(s,\pi_{d}^{SE},\phi_{a}^{*}\left(\pi_{d}^{SE}\right)\right)\geq J_{d}\left(s,\pi_{d},\phi_{a}^{*}\left(\pi_{d}\right)\right),\;\forall \pi_{d}\in \Pi_{d}.$$

where $\phi_{a}^{*}\left(\pi_{d}^{SE}\right)$ is expressed as (22).

**Proof.**  In the BSG, the channel gain and the strategy of the sensor can be obtained and imposed on the attacker; then, each type of attacker takes the best response to maximize its own payoff function. The sensor chooses the optimal strategy by taking account into the follower’s best response to maximize its own payoff functions, which completes the proof. □

Based on Lemma 1, the solution to the proposed Stackelberg game with incomplete channel gain information is given by the following theorem.

**Theorem** **1.** *The solution to the proposed BSG is given by first solving*

(24) $$\pi_{d}^{SE}=\phi_{d}^{*}\left(\phi_{a}^{*}\left(\pi_{d}^{SE}\right)\right),$$

*and then calculating*

(25) $$\begin{array}{c} \pi_{a}^{SE}=\left[\pi_{a}^{1,SE},\pi_{a}^{2,SE},\ldots ,\pi_{a}^{H,SE}\right], \end{array}$$

*where*

(26) $$\pi_{a}^{j,SE}=\phi_{a}^{j,*}\left(\pi_{d}^{SE}\right),j\in N.$$

*Therefore, the SE of this game is $\left(\pi_{d}^{SE},\pi_{a}^{SE}\right)\in \Pi_{d}\times \Pi_{a}$.*

**Proof.**  In the proposed BSG, the sensor as the leader and the DoS attacker as the follower make decisions sequentially. Given the sensor’s strategy $\pi_{d}$, the *j*-th type attacker will take the best response according to $\pi_{a}^{j}=\phi_{a}^{j,*}\left(\pi_{d}\right)$. The sensor knows the reaction from the attacker; hence, it will choose the optimal strategy to maximize its own payoff function while taking account into the attacker’s strategy, that is,

$$\begin{array}{cc} \pi_{d}^{SE} & =\arg\max_{\pi_{d}\in \Pi_{d}}J_{d}\left(s,\pi_{d},\phi_{a}^{*}\left(\pi_{d}^{SE}\right)\right)\\ & =\phi_{d}^{*}\left(\phi_{a}^{*}\left(\pi_{d}^{SE}\right)\right). \end{array}$$

After obtaining the sensor’s strategy $\pi_{d}^{SE}$, the *j*-th type attacker will choose the optimal strategy $\pi_{a}^{j,SE}$ by calculating

$$\pi_{a}^{j,SE}=\phi_{a}^{j}\left(\pi_{d}^{SE}\right),j\in N$$

which completes the proof. □

**Remark** **3.** *This theorem provides a constructive method to find the SE for the proposed BSG. It confirms that within the finite strategy spaces considered, the sensor can determine its optimal strategy by anticipating and incorporating the attacker’s best response, which, in turn, is computed based on the sensor’s declared action. This fixed-point characterization forms the theoretical foundation for the learning algorithm developed in Section 6.*

**Proposition** **1.** *There exists at least one SE in the BSG.*

**Proof.**  Given the sensor’s strategy $\pi_{d}$, the *j*-th type attacker will choose the strategy from the set $\pi_{a}^{j}$. Given the final state *K*, the numbers of possible strategies for the sensor and the *j*-th type attacker are $l_{d}^{K+1}$ and $l_{a}^{j,K+1}$, respectively. The *j*-th type attacker’s strategy space $\pi_{a}^{j}$ is finite, its optimal response $\phi_{a}^{i,*}\left(\pi_{d}\right)$ always exists for the fixed sensor’s strategy $\pi_{d}$. Moreover, the finite strategy space implies the existence of an equilibrium strategy for the sensor by (24). Therefore, there always exists an SE for the proposed BSG. □

**Remark** **4.** *This conclusion substantiates the existence and characterization of the SE in our Bayesian game setting. It implies that despite the incomplete information regarding the attacker’s type, an optimal hierarchical strategy profile exists and can be sought through iterative best-response dynamics.*

## 6. Reinforcement Learning

As mentioned previously, the sensor is unable to access to the SINR of the AWGN channel. In this section, model-free reinforcement learning, i.e., Q-learning is introduced to find the SE. Then, a Stackelberg Q-learning algorithm for the two-player BSG is used to obtain the optimal policy for both the sensor and the attacker.

The optimal payoff functions are given as

(27) $$\begin{array}{cc} J_{d}^{*}\left(s\right) & =\max_{\pi_{d}\in \Pi_{d}}\min_{\pi_{a}\in R_{a}\left(\pi_{d}\right)}Q_{d}^{*}\left(s,\lambda ,\delta \right), \end{array}$$

(28) $$\begin{array}{cc} J_{a}^{j,*}\left(s\right) & =\max_{\pi_{a}^{j}\in R_{a}^{j}\left(\pi_{d}^{SE}\right)}Q_{a}^{j,*}\left(s,\lambda^{SE},\delta^{j}\right), \end{array}$$

where the optimal state-action-value functions (Q-functions) for the sensor and the *j*-th type attacker are, respectively, defined as follows:

(29) $$\begin{array}{cc} Q_{d}^{*}\left(s,\lambda ,\delta \right) & =r_{d}\left(s,\lambda ,\delta \right)+\rho \sum_{s^{\prime }\in S}\left(\sum_{j=1}^{H}\eta \left(\beta_{j}\right)\mathrm{Pr}\left(s^{\prime }|s,\lambda ,\delta^{j}\right)\right)J_{d}^{*}\left(s^{\prime }\right),\\ Q_{a}^{j,*}\left(s,\lambda ,\delta^{j}\right) & =r_{a}^{j}\left(s,\lambda ,\delta^{j}\right)+\rho \sum_{s^{\prime }\in S}\mathrm{Pr}\left(s^{\prime }|s,\lambda ,\delta^{j}\right)J_{a}^{j,*}\left(s^{\prime }\right). \end{array}$$

Then, the SE strategy is obtained by

(30) $$\begin{array}{cc} \pi_{d}\left(s\right) & =\arg\max_{\pi_{d}\in \Pi_{d}}\min_{\pi_{a}\in R_{a}\left(\pi_{d}\right)}Q_{d}^{*}\left(s,\lambda ,\delta \right), \end{array}$$

(31) $$\begin{array}{cc} \pi_{a}^{j}\left(s\right) & =\arg\max_{\pi_{a}^{j}\in R_{a}^{j}\left(\pi_{d}^{SE}\right)}Q_{a}^{j,*}\left(s,\lambda^{SE},\delta^{j}\right). \end{array}$$

Considering the multi-type channel interference gain of the attacker, the Q-value functions of the sensor and the attacker are recursively updated by SE presented in Theorem 1:

(32) $$\begin{array}{cc} & Q_{k+1,d}\left(s,\lambda ,\delta \right)\\ = & \left(1-\alpha_{k}\right)Q_{k,d}\left(s,\lambda ,\delta \right)+\alpha_{k}\Psi_{k}\left(Q_{k,d}\left(s,\lambda ,\delta \right)\right), \end{array}$$

(33) $$\begin{array}{cc} & Q_{k+1,a}^{j}\left(s,\lambda ,\delta^{j}\right)\\ = & \left(1-\alpha_{k}\right)Q_{k,a}^{j}\left(s,\lambda ,\delta^{j}\right)+\alpha_{k}\Psi_{k}\left(Q_{k,a}^{j}\left(s,\lambda ,\delta^{j}\right)\right), \end{array}$$

where

(34) $$\begin{array}{cc} \Psi_{k}\left(Q_{k,d}\left(s,\lambda ,\delta \right)\right)\; & =r_{d}\left(s,\lambda ,\delta \right)+\rho \sum_{s^{\prime }\in S}\left(\sum_{j=1}^{H}\eta \left(\beta_{j}\right)\mathrm{Pr}\left(s^{\prime }|s,\lambda ,\delta^{j}\right)\right)stackelbergQ_{k,d}\left(s^{\prime },\lambda^{\prime },\delta^{\prime }\right), \end{array}$$

(35) $$\begin{array}{cc} \Psi_{k}\left(Q_{k,a}^{j}\left(s,\lambda ,\delta^{j}\right)\right)\; & =r_{a}^{j}\left(s,\lambda ,\delta^{j}\right)+\rho \sum_{s^{\prime }\in S}\mathrm{Pr}\left(s^{\prime }|s,\lambda ,\delta^{j}\right)stackelbergQ_{k,a}^{j}\left(s^{\prime },\lambda^{\prime },\delta^{j{,}^{\prime }}\right) \end{array}$$

with the learning rate $\alpha_{k}\in \left[0,1\right)$, $stackelbergQ_{k,d}\left(s^{\prime },\lambda^{\prime },\delta^{\prime }\right)$ and $stackelbergQ_{k,a}^{j}\left(s^{\prime },\lambda^{\prime },\delta^{j{,}^{\prime }}\right)$ are the Q-values of of the SE solutions at state $s^{\prime }$ for the sensor and the *j*-th type attacker, respectively.

In the BSG, the sensor and the attacker acts as the leader and the follower, respectively; hence, the update of Stackelberg Q-value is also hierarchically updated. To find the maximum Q-value of the attacker for the state $s^{\prime }$, the attacker chooses the optimal action from the best response (26). Based on the observation of the sensor’s action, the optimal action that the *j*-th type attacker takes in response to the sensor’s action $\lambda_{k}$ is determined as

(36) $$\phi_{a}^{j,*}\left(\lambda^{\prime }\right)=\arg\max_{\delta^{j}}Q_{k,a}^{j}\left(s^{\prime },\lambda^{\prime },\delta^{j}\right).$$

Then the optimal action of the attacker is given as

(37) $$\phi_{a}^{*}\left(\lambda^{\prime }\right)=\left[\phi_{a}^{1,*}\left(\lambda^{\prime }\right),\ldots ,\phi_{a}^{H,*}\left(\lambda^{\prime }\right)\right].$$

Based on the BSG framework, the optimal action of the sensor is given by

(38) $$\lambda^{*}=\arg\max_{\lambda^{\prime }}Q_{k,d}\left(s^{\prime },\lambda^{\prime },\phi_{a}^{*}\left(\lambda^{*}\right)\right),$$

Consequently, we compute $stackelbergQ_{k,d}\left(s^{\prime },\lambda^{\prime },\delta^{\prime }\right)$ and $stackelbergQ_{k,a}^{j}\left(s^{\prime },\lambda^{\prime },\delta^{j{,}^{\prime }}\right)$ in the Q-functions update as

(39) $$\begin{array}{cc} stackelbergQ_{k,d}\left(s^{\prime },\lambda^{\prime },\delta^{\prime }\right)\; & =Q_{k,d}\left(s^{\prime },\lambda^{*},\phi_{a}^{*}\left(\lambda^{*}\right)\right) \end{array}$$

(40) $$\begin{array}{cc} stackelbergQ_{k,a}^{j}\left(s^{\prime },\lambda ,\delta^{j{,}^{\prime }}\right)\; & =Q_{k,a}^{j}\left(s^{\prime },\lambda^{*},\phi_{a}^{j,*}\left(\lambda^{*}\right)\right) \end{array}$$

As a result, the Bayesian Stackelberg Q-learning Algorithm for the incomplete channel gain is presented in Algorithm 1. Key implementation details are as follows: The Q-values for both players are initialized to zero, a common practice that does not bias the asymptotic convergence. This initialization does not affect the final optimal policy learned, ensuring the algorithm’s robustness to initial conditions. An ϵ-greedy exploration strategy is employed, where, with probability ϵ, the actions are chosen randomly, and with probability 1−ϵ, they are chosen as the SE actions based on current Q-values. The learning rate $\alpha_{k}$ for updating the Q-tables follows a schedule that ensures the Robbins-Monro conditions are met (as required by Theorem 2), typically by decaying with the number of visits to each state-action pair. The specific forms of $\alpha_{k}$ and ϵ used in our simulations are provided in Section 7.
**Algorithm 1** Bayesian Stackelberg Q-learning Algorithm with Incomplete Channel Gain Information**Input:** System parameter matrices *A*, *C*, *Q*, *R*, $\sigma^{2}$   1:
   2:Initialization: Initialize $Q_{0,d}\left(s,\lambda ,\delta \right)$ and $Q_{0,a}^{j}\left(s,\lambda ,\delta^{j}\right)$ for all j∈N, s∈S, λ∈Λ, and $\delta^{j}\in \Delta^{j}$; initial state $s_{0}$; Total iterations *T*; exploration probability ε. Set iteration counter k=1;   3:**while** k<T**do**   4:      Randomize a number ζ∈[0,1];   5:      **if** ζ∈[ε,1] **then**   6:          Take actions λ and $\delta^{j}$ for j∈N based on (36)–(38);   7:      **else**   8:          Select random actions λ and $\delta^{j}$ for j∈N;   9:      **end if** 10:      Obtain rewards $r_{d}\left(s,\lambda ,\delta \right)$ and $r_{d}\left(s,\lambda ,\delta \right)$; 11:      Update $Q_{k+1,d}\left(s,\lambda ,\delta \right)$ and $Q_{k+1,a}^{j};\left(s,\lambda ,\delta^{j}\right)$, and move to next state; 12:      k=k+1; 13:**end while****Output:** The optimal strategies and the optimal payoff functions for the sensor and the attacker

**Remark** **5.** *The per-iteration computational cost of the Stackelberg Q-learning algorithm (Algorithm 1) is linear in the product of the state space size |S| and the action space sizes $\left|\Lambda |\times \right|\Delta^{j}|$, as it requires updating Q-tables for all state–action pairs encountered. This makes it efficient for the moderately sized problems presented here. For systems with significantly larger state or action spaces, the well-known curse of dimensionality would necessitate the use of function approximation (e.g., deep Q-networks), which is a promising direction for future work, as noted in Section 8.*

The convergence of Algorithm 1 to the optimal Q-functions is guaranteed under the conditions specified in Theorem 2. The learning rate and exploration schedules described above are designed to satisfy these conditions, in particular the Robbins–Monro requirements for the learning rate.

**Theorem** **2** **([36]).** *The Bayesian Stackelberg Q-learning sequences described in* (32) *and* (33) *will converge to the optimal values if the following assumptions hold:*

*1.*  *The recursive processes* (32) *and* (33) converge to $Q_{d}{\left(s,\lambda ,\delta \right)}^{*}$ and $Q_{a}{\left(s,\lambda ,\delta \right)}^{*}$ with probability 1, respectively.
*2.*  *There exists a number a∈(0,1) and a sequence $\varsigma_{k}\geq 0$ converging to zero with probability 1, such that*
$$\begin{array}{cc} ∥\Psi_{k}\left(Q_{d}\right)-\Psi \left(Q_{d}^{*}\right)∥ & \leq a∥Q_{d}-Q_{d}^{*}∥+\varsigma_{k},\forall Q_{d}\in Q\\ ∥\Psi_{k}\left(Q_{a}^{j}\right)-\Psi \left(Q_{a}^{j,*}\right)∥ & \leq a∥Q_{a}^{j}-Q_{a}^{j,*}∥+\varsigma_{k},\forall Q_{a}\in Q \end{array}$$
  *where Q is the Q-value space.*
*3.*  *The learning rate $\alpha_{k}$ satisfies that $\alpha_{k}\in \left[0,1\right)$, and $\sum_{k=1}^{n}\alpha_{k}$ converges to infinity uniformly as n→∞.*

**Remark** **6.** *Condition 1 assumes that the recursive updates for both players converge with probability 1, meaning the learning process is inherently stable and reaches a fixed point. This is approximated by implementing ε-greedy exploration in Algorithm 1 and running the training for a large number of steps. Condition 2 ensures that the algorithm is driven toward a unique fixed point while noise vanishes by a contraction operator with a decaying perturbation. The learning rate in Condition 3 must satisfy the Robbins–Monro conditions for stochastic approximation. In the simulation section, the learning rate $\alpha_{k}$ is designed to be a nonzero decreasing function of time step k and the current state and actions.*

## 7. Simulation Results

In this section, a numerical example is provided to verify the performance of the proposed stochastic attack strategy. The following system parameter are considered:

$$\begin{array}{cc} A & =\left[\begin{array}{cc} 1 & 0.5\\ 0 & 1.05 \end{array}\right],\;C=\left[\begin{array}{cc} 1 & 0 \end{array}\right],\;Q=0.5*I_{2},\;R=0.5. \end{array}$$

Then the steady-state error covariance $\bar{P}$ is $\bar{P}=\left[0.3802,0.2840;0.2840,1.6894\right]$. The AWGN power and the modulation parameter are $\sigma_{k}=0.5$ and κ=0.2, respectively. The fading channel gain and the action space of the sensor are given as α=0.7 and $A_{d}=\left\{1,2,3\right\}$. Assume that the fading channel gains of the DoS attacker are [0.5,0.8] with probability distribution [0.8,0.2]. This setting creates a representative asymmetric information scenario in which the sensor needs to design a strategy against an attacker that is most likely to have a moderate interference capability but must also account for a significant possibility of facing a more potent attacker. Correspondingly, the action spaces for the two types of the attacker are $A_{a}^{1}=\left\{4,5,6\right\}$ and $A_{a}^{2}=\left\{1,2,3\right\}$, respectively. The unit power costs for the sensor and the attacker are $C_{d}=1$ and $C_{a}=2$, respectively. Set the finial state to K=4, and the state space is given as S={0,1,2,3,4}, which means the estimation error takes the value from $\left\{\bar{P},h\left(\bar{P}\right),\ldots ,h^{4}\left(\bar{P}\right)\right\}$. The discount factor for payoff functions is ρ=0.9.

In the Bayesian Stackelberg Q-learning algorithm, the learning rate and the initial exploring rate are set as α=10/[15+count(s,λ,δ)] and $\epsilon_{0}=0.98$, respectively, where count(s,λ,δ) is the number of the occurrence of state–action pair (s,λ,δ). This design ensures that the learning rate satisfies the Robbins–Monro conditions, a standard requirement for the convergence of stochastic approximation algorithms like Q-learning. The initial values are set as $Q_{0,d}\left(s,\lambda ,\delta \right)=0$ and $Q_{0,a}^{j}\left(s,\lambda ,\delta^{j}\right)=0$ for all j∈N.

After 100,000 learning steps, the Q function values in different states converge. The equilibrium payoffs to the sensor and attacker for states $s=\bar{P},\ldots ,h^{4}\left(\bar{P}\right)$ are given as follows:

$$\begin{array}{cc} J_{d}^{*}\left(s\right) & =[-131.9945,-141.6237,-149.8064,-155.1217,-155.1217],\\ J_{a}^{1,*}\left(s\right) & =[81.5995,87.9412,95.6919,101.0546,101.0546],\\ J_{a}^{2,*}\left(s\right) & =[97.7494,103.6945,111.4222,116.5348,116.5348]. \end{array}$$

The corresponding optimal transmission and interference strategies are given as follows:

$$\begin{array}{cc} \pi_{d}^{*}\left(s\right) & =[1,3,3,3,3],\\ \pi_{a}^{1,*}\left(s\right) & =[4,4,5,6,6],\\ \pi_{a}^{2,*}\left(s\right) & =[1,3,3,3,3]. \end{array}$$

The resulting Stackelberg equilibrium strategies are explicitly state-dependent, quantitatively showing how the sensor optimally increases transmission power as the estimation error grows. Finally, the equilibrium payoffs are quantitatively characterized, showing the cost–performance trade-off across states. The optimal strategies under the BSG confirm the intuition that a relatively small error covariance at the remote estimator enables the sensor to use less power for transmission, also leading to less interference power for the attacker. For a larger estimation error covariance, the sensor is inclined to choose a high power level to increase the packet arrival rate and, therefore, improve estimation performance. In this case, the attacker also increases the interference power accordingly.

The algorithm converges to distinct, stable Q-values for different state–action pairs. Due to the high dimension of the Q-values $Q_{k}\left(s,\lambda ,\delta \right)$, next, we present the learning process for the initial state $s=\bar{P}$ as an illustrative example. When the sensor takes the action λ=1, the corresponding Q-functions converge, as shown in Table 1. Table 2 gives all converged Q-values of the attacker. We can see that the optimal strategy for the attacker is δ=(4,1) under state $s=\bar{P}$.

**Figure 2:**
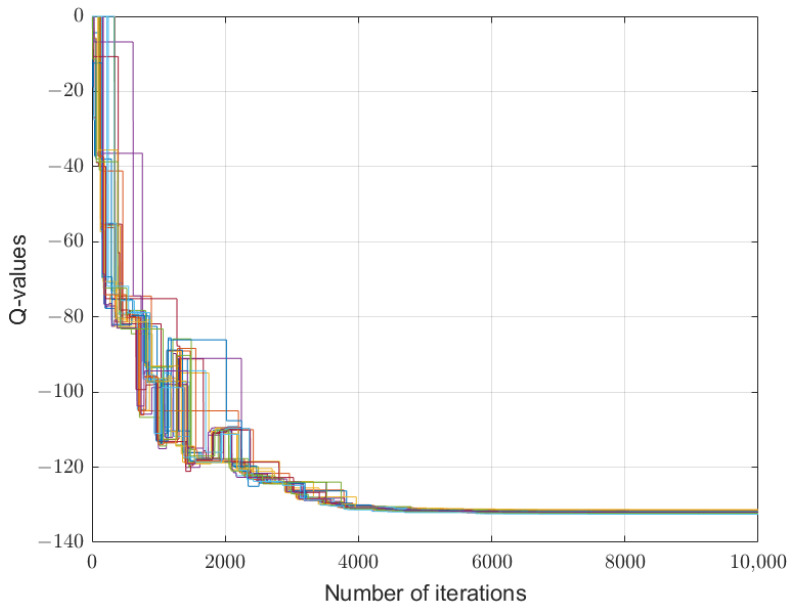
Convergence of Q-values for the sensor at state $\bar{P}$. The multitude of colored lines represents the learning trajectories of $Q_{k,d}\left(s,\lambda ,\delta \right)$ for all possible combinations of the sensor’s transmission power λ and the attacker’s interference power $\delta =(\delta^{1},\delta^{2})$. The collective convergence of all lines after approximately 5000 iterations demonstrates the stabilization of the learning process. The specific equilibrium Q-values that define the optimal strategy are listed in Table 1.

**Figure 3:**
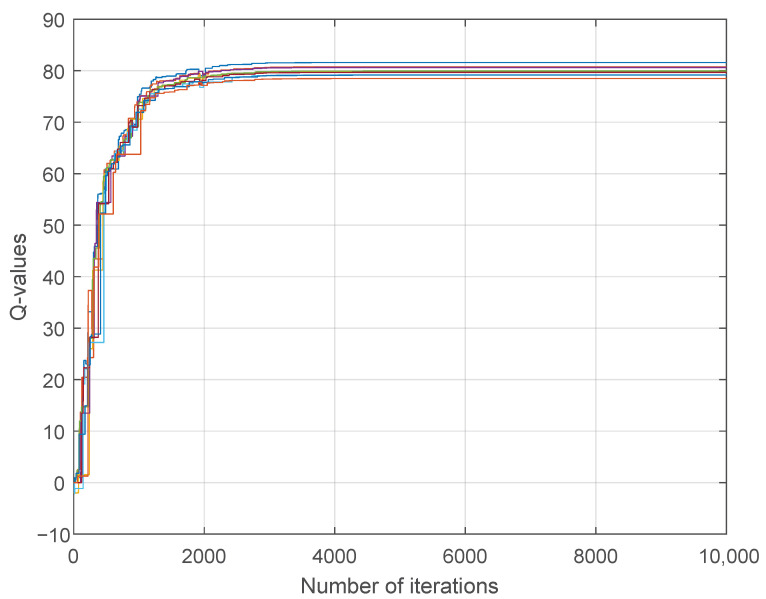
Convergence of Q-values for the first-type attacker at state $\bar{P}$. Each colored line corresponds to the learning trajectory of $Q_{k,a}^{1}\left(s,\lambda ,\delta^{1}\right)$ for a specific pair of sensor power and attacker power. The convergence of all lines illustrates the algorithm’s stability for this attacker type. The resulting equilibrium values are part of the dataset summarized in Table 2.

**Figure 4:**
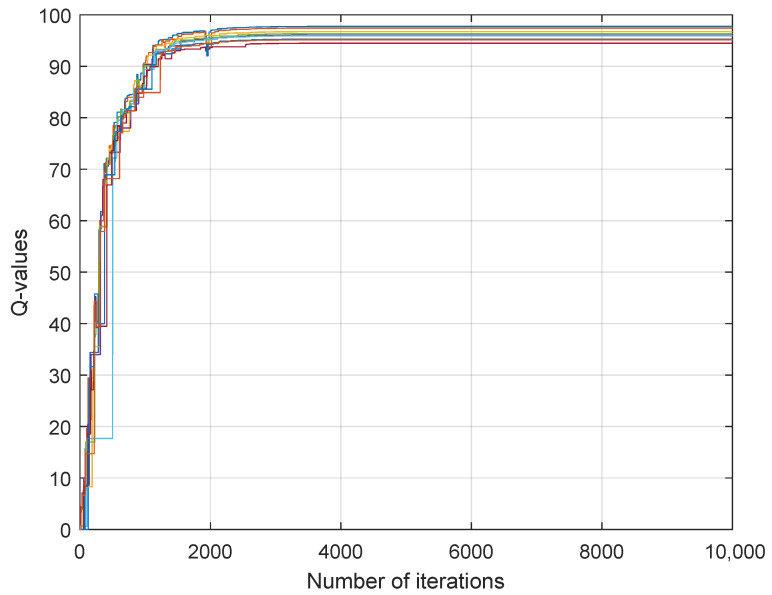
Convergence of Q-values for the second-type attacker at state $\bar{P}$. Each colored line corresponds to the learning trajectory of $Q_{k,a}^{2}\left(s,\lambda ,\delta^{2}\right)$ for a specific pair of sensor power and attacker power. The convergence of all lines illustrates the algorithm’s stability for this attacker type. The resulting equilibrium values are part of the dataset summarized in Table 2.

Figure 2, Figure 3 and Figure 4 depict the learning processes for the optimal strategies of the sensor, the first type attacker, and the second type attacker for state $\bar{P}$, respectively. In the figures, different colored lines respectively represent the iterative Q-values under different actions of the sensor and attacker. These figures collectively depict the convergence of Q-functions for all possible action combinations at state $\bar{P}$. The high density of lines illustrates the learning algorithm’s exploration across the entire strategy space. While individual lines are not labeled for clarity, their collective trend towards stabilization after around 5000 iterations is evident, confirming the convergence of the learning process. The precise converged Q-values that underpin the optimal strategies are detailed in Table 1 and Table 2.

Next, we consider the situation that the attacker can launch attacks without any cost constraints, that is, the cost of the interference power can be ignored. In the case of $C_{a}=0.01$, the equilibrium payoffs to the sensor and attacker for states $s=\bar{P},\ldots ,h^{4}\left(\bar{P}\right)$ are given as follows:

$$\begin{array}{cc} J_{d}^{*}\left(s\right) & =[-138.7763,-148.5242,-156.6659,-161.9812,-161.9812],\\ J_{a}^{1,*}\left(s\right) & =[189.0304,196.9475,205.1795,210.5454,210.5454],\\ J_{a}^{2,*}\left(s\right) & =[155.1126,162.1956,169.9786,175.0912,175.0912]. \end{array}$$

The corresponding optimal transmission and interference strategies are given as follows:

$$\begin{array}{cc} \pi_{d}^{*}\left(s\right) & =[1,3,3,3,3],\\ \pi_{a}^{1,*}\left(s\right) & =[6,6,6,6,6],\\ \pi_{a}^{2,*}\left(s\right) & =[3,3,3,3,3]. \end{array}$$

The optimal strategies confirm that when the cost of interference power $C_{a}$ is negligible, both types of attackers consistently select the highest available power level in their respective action sets. This behavior aligns perfectly with game-theoretic intuition: as the marginal cost of interference diminishes, the rational objective for the attacker shifts overwhelmingly towards maximizing the degradation of the estimation performance, leading to the selection of maximum jamming power. This result underscores the critical role of cost parameters in shaping the equilibrium of the security game.

**Figure 5:**
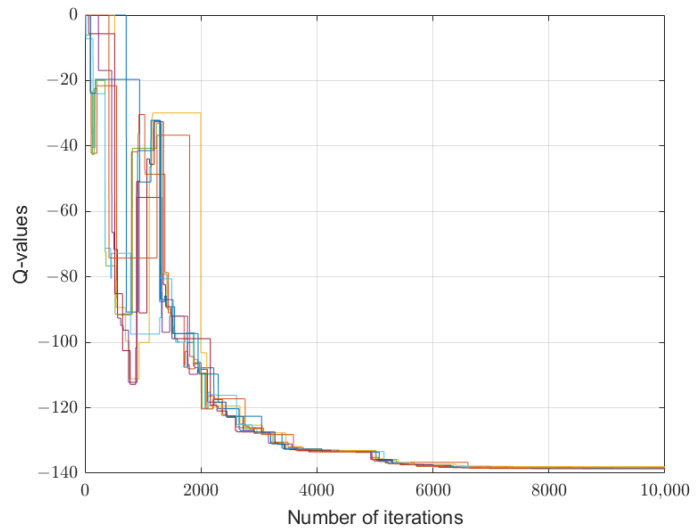
Convergence of Q-values for the sensor at state $\bar{P}$ under $C_{a}=0.01$. The multitude of colored lines represents the learning trajectories of $Q_{k,d}\left(s,\lambda ,\delta \right)$ for all possible combinations of the sensor’s transmission power λ and the attacker’s interference power $\delta =(\delta^{1},\delta^{2})$.

**Figure 6:**
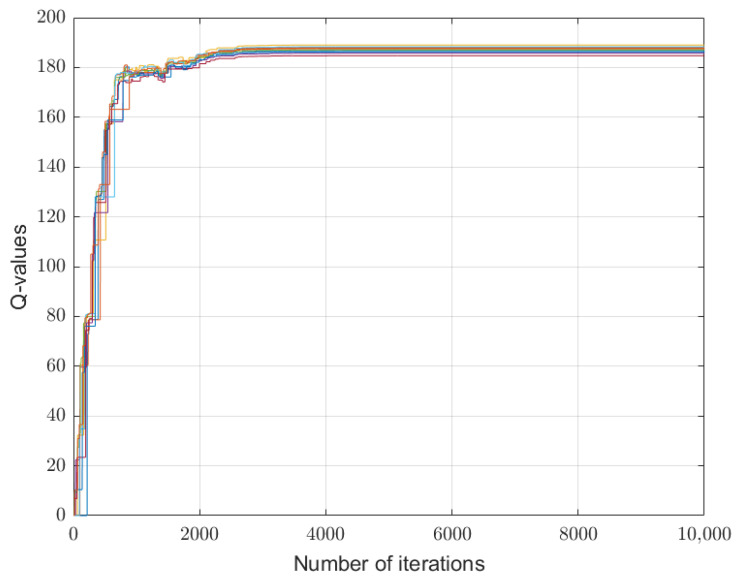
Convergence of Q-values for the first-type attacker at state $\bar{P}$ under $C_{a}=0.01$. Each colored line corresponds to the learning trajectory of $Q_{k,a}^{1}\left(s,\lambda ,\delta^{1}\right)$ for a specific pair of sensor power and attacker power. The convergence of all lines illustrates the algorithm’s stability for this attacker type.

**Figure 7:**
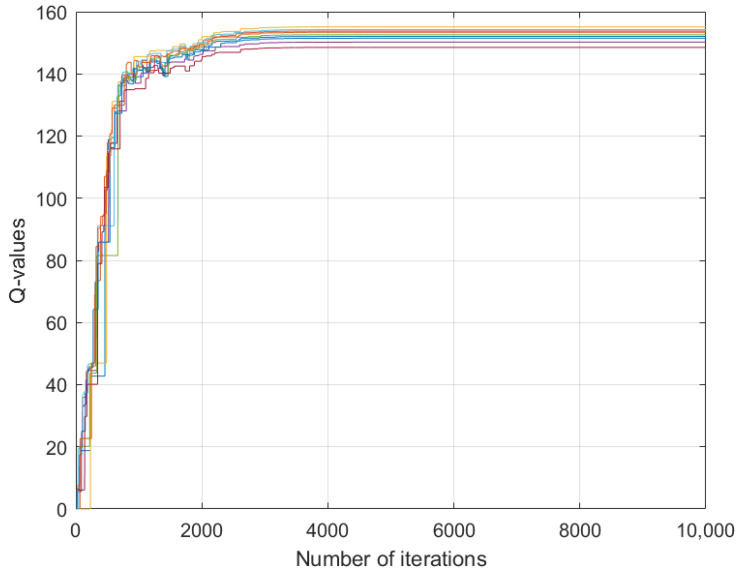
Convergence of Q-values for the second-type attacker at state $\bar{P}$ under $C_{a}=0.01$. Each colored line corresponds to the learning trajectory of $Q_{k,a}^{2}\left(s,\lambda ,\delta^{2}\right)$ for a specific pair of sensor power and attacker power. The convergence of all lines illustrates the algorithm’s stability for this attacker type.

The learning processes for the optimal strategies of the sensor, the first type attacker, and the second type attacker for state $\bar{P}$ are shown in Figure 5, Figure 6 and Figure 7. Similar to the previous case, the convergence trends for all action combinations are shown. The eventual stabilization of all trajectories validates the algorithm’s effectiveness even in this complex setting. Notably, as seen in Figure 5, the Q-values of the sensor exhibit significant fluctuation and converge more slowly compared to those of the attackers in Figure 6 and Figure 7. This can be attributed to the information asymmetry inherent in the problem: the sensor lacks precise knowledge of the attacker’s channel gain and must learn an optimal strategy based only on the probabilistic distribution over attacker types. This uncertainty inherently increases the exploration burden and complexity of the learning process for the leader.

Therefore, the numerical results validate the algorithm’s effectiveness by demonstrating its capability to solve the proposed BSG model: It is seen that the algorithm achieves convergence to a stable equilibrium where both players’ strategies are mutually optimal in the sequential sense, as theorized in Section 5 and Section 6. The logical adaptation of strategies to different states and costs further confirms that the learned policies align with game-theoretic intuition, providing strong empirical support for the proposed framework.

## 8. Conclusions

This paper investigated the Bayesian Stackelberg game for remote state estimation under SINR-based DoS attacks. The two players sequentially decide their transmission and interference powers under incomplete information. Specifically, the sensor lacks exact knowledge of the attacker’s fading channel gain. The optimization problem over an infinite-time horizon is first formulated as an MDP with finite state and action spaces. By taking advantage of the probabilistic information about the channel interference gain, a BSG is modeled to describe the iterative decision-making process between the sensor and the various types of attackers. Based on the solution to the BSG, a Stackelberg Q-learning algorithm is used to obtain the optimal strategies of the two players. Numerical results validate the effectiveness of the proposed game-theoretic approach in the case of uncertain channel gains. The presented framework operates under core assumptions of a known attacker type distribution and discrete action spaces, which define its current scope but also present opportunities for future generalization. Future work also includes analyzing games where both players have incomplete information and extending the framework to larger-scale systems via function approximation (e.g., deep reinforcement learning) to mitigate the curse of dimensionality.

## Figures and Tables

**Table 1:** Converged Q-values $Q_{d}\left(s,\lambda ,\delta \right)$ of the sensor for state $s=\bar{P}$ and sensor action λ=1. The columns represent the joint attacker actions $\delta =(\delta^{1},\delta^{2})$.

δ	$Q_{d}\left(s,\lambda ,\delta \right)$
(4,1)	−131.5542
(5,1)	−131.6878
(6,1)	−131.7834
(4,2)	−131.6996
(5,2)	−131.8332
(6,2)	−131.9288
(4,3)	−131.7653
(5,3)	−131.8989
(6,3)	−131.9945

**Table 2:** Converged Q-values for both attacker types at state $s=\bar{P}$. Each row corresponds to a sensor action λ, with columns showing the Q-values for attacker type 1 ($Q_{d}^{1}$ under action $\delta^{1}$) and type 2 ($Q_{d}^{2}$ under action $\delta^{2}$).

λ	$\delta^{1}$	$\delta^{2}$	$Q_{a}^{1}\left(s,\lambda ,\delta^{1}\right)$	$Q_{a}^{2}\left(s,\lambda ,\delta^{2}\right)$
1	4	1	81.5995	97.7494
1	5	2	80.6239	96.0405
1	6	3	79.6724	94.4824
2	4	1	80.7636	97.4259
2	5	2	79.9470	96.3323
2	6	3	79.1444	95.2768
3	4	1	79.8811	96.7319
3	5	2	79.1792	95.9321
3	6	3	78.4862	95.1471

## Data Availability

The original contributions presented in this study are included in the article.
